# Role of the Two Flagellar Stators in Swimming Motility of Pseudomonas putida

**DOI:** 10.1128/mbio.02182-22

**Published:** 2022-11-21

**Authors:** Veronika Pfeifer, Sönke Beier, Zahra Alirezaeizanjani, Carsten Beta

**Affiliations:** a Institute of Physics and Astronomy, University of Potsdam, Potsdam, Germany; Max Planck Institute for Terrestrial Microbiology

**Keywords:** bacterial swimming, stators, structured environments

## Abstract

In the soil bacterium Pseudomonas putida, the motor torque for flagellar rotation is generated by the two stators MotAB and MotCD. Here, we construct mutant strains in which one or both stators are knocked out and investigate their swimming motility in fluids of different viscosity and in heterogeneous structured environments (semisolid agar). Besides phase-contrast imaging of single-cell trajectories and spreading cultures, dual-color fluorescence microscopy allows us to quantify the role of the stators in enabling P. putida’s three different swimming modes, where the flagellar bundle pushes, pulls, or wraps around the cell body. The MotAB stator is essential for swimming motility in liquids, while spreading in semisolid agar is not affected. Moreover, if the MotAB stator is knocked out, wrapped mode formation under low-viscosity conditions is strongly impaired and only partly restored for increased viscosity and in semisolid agar. In contrast, when the MotCD stator is missing, cells are indistinguishable from the wild type in fluid experiments but spread much more slowly in semisolid agar. Analysis of the microscopic trajectories reveals that the MotCD knockout strain forms sessile clusters, thereby reducing the number of motile cells, while the swimming speed is unaffected. Together, both stators ensure a robust wild type that swims efficiently under different environmental conditions.

## INTRODUCTION

Flagella-mediated swimming is one of the most common strategies of locomotion in the bacterial world. Powered by membrane-embedded molecular motors, bacteria rotate their flagella to propel themselves and have evolved a wide variety of swimming strategies, depending on the arrangement of flagella across the cell body (1). The flagellar movement is driven by stator complexes that rely on ion gradients across the membrane to generate the torque required for rotation. This machinery is dynamic, with stators being constantly recruited and released from the motor, exchanging with a large pool of freely diffusing stators in the membrane so that bacteria can flexibly adapt to changing environmental conditions (2). The widely studied model organism Escherichia coli operates a single type of stator, the proton-driven MotAB complex. It consists of a MotB dimer surrounded by five MotA proteins (3, 4). The stator recruitment is mechanosensitive. Thereby, the flagellar motor adapts to changes in viscous load, and bacteria can navigate through local inhomogeneities and obstacles (5–7). Depending on the mechanical load on the flagellum, up to 11 MotAB stators can bind to the motor of E. coli (8, 9). However, numerous bacteria have more than one type of stator, allowing them to fine-tune their motor function by, for example, changing the stator composition and recruiting the more favorable stator for a given task. Shewanella oneidensis has sodium-driven PomAB stators and proton-driven MotAB stators to adjust its motor function in the presence of different sodium concentrations (10). In Vibrio parahaemolyticus, two flagellar systems with two different types of stators have evolved. The polar flagellum is propelled by sodium-driven stators and the lateral flagella by proton-driven ones (11). In Pseudomonas aeruginosa, the two stators, MotAB and MotCD, use proton motive force to power the rotation of a single flagellum. Both stators can promote swimming motility in aqueous environments, but only MotCD can maintain swimming under high-viscosity conditions and swarming across surfaces (12, 13). Interestingly, the torque generated per stator unit is the same for MotAB and MotCD. However, the total torque produced by MotAB during swimming in aqueous environments is higher because, on average, more MotAB stators are recruited to the motor (14).

The closely related soil bacterium Pseudomonas putida also possesses the two stators, MotAB and MotCD (15). However, P. putida does not swarm in a flagella-dependent manner (16) and is lophotrichously flagellated, having multiple flagella at one cell pole (17). P. putida displays a complex swimming pattern consisting of straight runs with two alternating speeds that are interrupted by stops and directional reversals (18). Three different run modes can be distinguished. When the flagellar bundle rotates counterclockwise (CCW), it pushes the cell body forward. When it rotates clockwise (CW), it can either pull the cell body or wrap around it. The latter swimming mode results in a slower swimming speed (19). Moreover, in the wrapped mode, P. putida responds to chemoattractant gradients, which is considered to be a beneficial strategy to navigate its crowded natural habitat (20). The wrapped mode is known to also form in other polarly flagellated species (21), such as monopolarly flagellated Shewanella putrefaciens (22) and lophotrichously flagellated *Burkholderia* sp. strain RPE64 (23). More recently, it was also discovered in amphitrichous Campylobacter jejuni (24) and monotrichous P. aeruginosa (25).

For P. putida, the question arises of whether there is a specific role of the two different stators, MotAB and MotCD, for swimming motility and how the stators influence the swimming modes, especially the formation of the wrapped mode. It is assumed that the wrapped mode is formed due to changes in motor torque (19, 22). Recently, numerical simulations supported this conjecture, showing increased wrapped mode formation under higher torque (26). In this study, we knocked out the two stators, MotAB and MotCD, and analyzed swimming motility under different environmental conditions to address these questions.

## RESULTS

### Construction of stator deletion mutants.

P. putida has a large gene cluster for flagellar motility comprising the genes for the stator MotCD (PP4336 and PP4335). Additionally, the genome encodes a second copy of a stator outside this gene cluster, the MotAB (PP4905 and PP4904) (15). Protein sequence alignments show a high similarity to the MotAB and MotCD stators in P. aeruginosa PAO1 (see Fig. S1 in the supplemental material). MotA shares 81% identity, MotB shares 70% identity, MotC shares 83% identity, and MotD shares 70% identity. We targeted these genes in P. putida to generate single and double deletion mutants. As a control, we rescued the mutants by reintroducing the genes. Swimming in aqueous environments and swimming agar assays confirmed a fully restored wild-type phenotype (Fig. S2). For none of the mutants was the growth rate influenced, so all cultures grew at a similar speed.

10.1128/mbio.02182-22.1 FIG S1 Protein sequence alignment for the stators MotAB and MotCD in P. putida and P. aeruginosa. Alignment was made with BLAST (https://blast.ncbi.nlm.nih.gov/). Download FIG S1, PDF file, 0.5 MB. Copyright © 2022 Pfeifer et al. 2022 Pfeifer et al. https://creativecommons.org/licenses/by/4.0/ This content is distributed under the terms of the Creative Commons Attribution 4.0 International license.

10.1128/mbio.02182-22.2 FIG S2 Complementation analysis for *motAB* and *motCD*. (A) Complementation of MotAB leads to a very motile phenotype in aqueous environments. The swimming motility of *ΔmotAB* motAB cells is comparable with the wild-type swimming motility. (B) Complementation of MotCD in the *ΔmotCD* mutant leads to increased spreading in agar comparable to the spreading of wild-type cells. Due to complementation of MotAB in the *ΔmotAB ΔmotCD* double mutant, the strain shows a similar phenotype to the *ΔmotCD* mutant. Download FIG S2, PDF file, 0.2 MB. Copyright © 2022 Pfeifer et al. 2022 Pfeifer et al. https://creativecommons.org/licenses/by/4.0/ This content is distributed under the terms of the Creative Commons Attribution 4.0 International license.

### MotAB is essential for swimming and wrapped mode formation under low-viscosity conditions.

**FIG 1:**
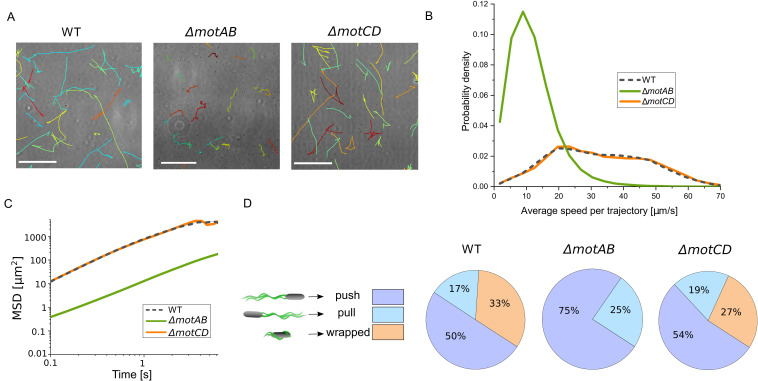
Swimming in an aqueous environment reveals strong deficiencies of the *ΔmotAB* mutant, whereas the *ΔmotCD* mutant shows similar behavior to the wild-type strain. (A) Swimming trajectories for the wild type and the *ΔmotAB* and *ΔmotCD* mutant strains. Scale bar, 100 μm. (B) Distributions of mean speeds per trajectory. (C) Mean square displacement over time. (D) Pie charts showing the ratios of the different swimming modes contributing to swimming motility. We manually analyzed 441 runs for the wild-type strain, 216 runs for the *ΔmotAB* mutant, and 268 runs for the *ΔmotCD* mutant. The cartoons on the left show the three swimming modes, push, pull, and wrapped; the arrows indicate the swimming direction.

To characterize the role of the two stators, we compared the swimming motility of the wild type, the single mutants *ΔmotAB* and *ΔmotCD*, and the double mutant *ΔmotAB ΔmotCD*. A visual inspection of swimming motility from shaking cultures with phase-contrast microscopy revealed clear differences between the four strains. The double mutant *ΔmotAB ΔmotCD* is nonmotile. Swimming motility of the *ΔmotCD* mutant is indistinguishable from the swimming motility of wild-type cells, whereas the *ΔmotAB* mutant shows strong deficiencies in swimming. Figure 1A shows a selection of the longest trajectories resulting from cell segmentation and tracking with a custom-made MATLAB software. While for the wild type and the *ΔmotCD* strain, the trajectories mainly consist of long, straight runs that are interrupted by occasional abrupt turning events, the *ΔmotAB* strain exhibits only short, erratic trajectories. In Fig. 1B, the distributions of the mean swimming speeds per trajectory are shown. When calculating the mean speed over the complete set of trajectories, we obtained 32.1 μm/s ± 0.3 μm/s for the wild type, 6.6 μm/s ± 0.2 μm/s for the *ΔmotAB* mutant, and 32.5 μm/s ± 0.3 μm/s for the *ΔmotCD* mutant. Thus, the *ΔmotAB* mutant swims more than four times more slowly than the wild type, which is also reflected in a shift of the mean square displacement (MSD) toward lower values of the diffusivity; see Fig. 1C. In order to distinguish between the three different run modes—push, pull, and wrapped—that are part of P. putida’s swimming strategy, we stained the flagella and visualized them by fluorescence microscopy with high spatial and temporal resolution. Figure 1D shows the frequency of the three run modes as they were observed during swimming of the wild type, the *ΔmotAB*, and the *ΔmotCD* mutant strains. In agreement with the morphology of the trajectories observed by phase-contrast imaging, the distributions of run modes for the wild type and the *ΔmotCD* mutant strains are similar, while the distribution for the *ΔmotAB* mutant differs. Not only are the *ΔmotAB* cells less motile in an aqueous environment but they are also not able to form the wrapped mode. Surprisingly, the ratio of CW (pull and wrap mode) to CCW (push mode) rotation of the flagella is influenced as well: the *ΔmotAB* mutant shows an increased amount of runs with CCW flagellar rotation.

### Increasing viscosity partly restores wrapped mode formation in the absence of MotAB.

For other wrapped mode-forming bacteria, it was shown that a more viscous surrounding medium leads to an increased portion of runs in the wrapped mode (22, 24). To test whether P. putida shows a similar behavior, we added Ficoll to swimming wild-type and stator mutant strains to increase the viscosity and, thus, the load on the flagella. In particular, it was our aim to investigate whether the MotAB stator is strictly indispensable for wrapped mode formation or whether there are conditions where also the *ΔmotAB* knockout mutant is able to form the wrapped mode.

**FIG 2:**
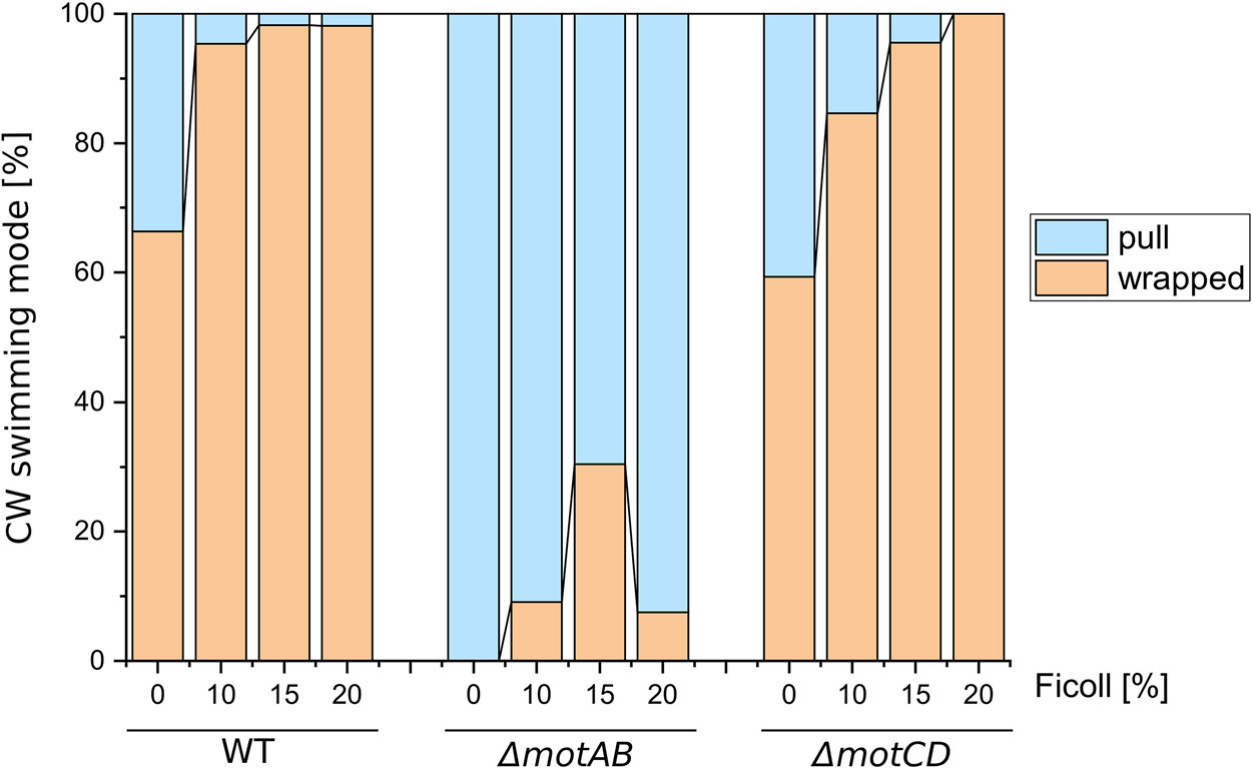
Influence of increased viscosity on the ratio of CW swimming modes, pull and wrapped. In general, the stepwise increase in viscosity led to increased wrapped mode formation; even *ΔmotAB* was able to form the wrapped mode under higher-viscosity conditions. However, for the *ΔmotAB* mutant, the amount of wrapped mode increased only for up to 15% Ficoll and decreased again for 20% Ficoll. The ratio of the three swimming modes, push, pull, and wrapped, and the sample sizes of all experiments are listed in Table S1 in the supplemental material.

In general, an increased Ficoll concentration led to decreased swimming speeds for all three strains (Table 1). The decrease in swimming speed for the *ΔmotCD* mutant was similar to the decrease in the wild-type strain. In contrast, the swimming speed of *ΔmotAB* cells was already much lower in the absence of Ficoll. Adding Ficoll slowed the cells down even further, so that for Ficoll concentrations of 15% and higher, it was no longer possible to extract any trajectories. Nevertheless, the swimming modes could be still identified by fluorescence microscopy. We determined the swimming motility and flagellar configuration after adding 10%, 15%, and 20% Ficoll. Under conditions of increased viscosity, the *ΔmotAB* mutant showed a CW-to-CCW ratio comparable to the wild type and the *ΔmotCD* mutant: for the Ficoll experiments, the CW-to-CCW ratio stays roughly constant for all strains, with the CCW push mode occurring between 50% and 65%; see Table S1. Figure 2 shows the distribution of the CW swimming modes, pull and wrap, at different Ficoll concentrations. For the wild-type and the *ΔmotCD* strains, the portion of wrapped runs increases, until at 20% Ficoll, almost all CW runs are taking place in the wrapped mode. Interestingly, also, the *ΔmotAB* mutant is able to form the wrapped mode under high-viscosity conditions. However, at 20% Ficoll, the amount of wrapped runs decreases again in contrast to the wild-type and the *ΔmotCD* cells.

**TABLE 1:** Mean swimming speeds in different environments^*a*^

Condition	Data for:					
	WT		*ΔmotAB*		*ΔmotCD*	
	Speed (μm/s)	No. of samples	Speed (μm/s)	No. of samples	Speed (μm/s)	No. of samples
0% Ficoll	32.1 ± 0.3	1,898	6.6 ± 0.2	280	32.5 ± 0.3	1,911
10 % Ficoll	11.4 ± 0.3	590	5.8 ± 1.5	7	11.6 ± 0.2	847
15% Ficoll	8.9 ± 0.2	379			7.7 ± 0.2	339
20% Ficoll	6.3 ± 0.6	52			6.3 ± 0.4	70
0.25% agar	23.85 ± 0.05^*b*^	27,799	21.44 ± 0.04^*b*^	43,126	27.18 ± 0.12^*b*^	6,889

a Speed values, unless otherwise specified, were calculated over the entire trajectories, including runs and turns, because in the case of the slowly swimming *ΔmotAB* cells, runs and turns cannot be distinguished. The smaller sample sizes for the *ΔmotAB* mutant in bulk experiments and the *ΔmotCD* mutant in agar experiments reflect the decreased amount of motile cells in comparison to the other two strains in the same environment. For the *ΔmotAB* mutant, motility was too low to extract the swimming speed at Ficoll concentrations of 15% or higher.

b Values were calculated from the run episodes only.

10.1128/mbio.02182-22.3 TABLE S1 Frequency of swimming modes for different Ficoll concentrations. For each experiment, the sample size is listed. For the *ΔmotAB* mutant, it is smaller due to the decreased motility. However, for 0% and 20% Ficoll, we analyzed a larger number of measurements to get a comparable sample size. Download Table S1, PDF file, 0.1 MB. Copyright © 2022 Pfeifer et al. 2022 Pfeifer et al. https://creativecommons.org/licenses/by/4.0/ This content is distributed under the terms of the Creative Commons Attribution 4.0 International license.

### In semisolid agar, the *ΔmotAB* mutant spreads similarly to the wild type and outperforms the *ΔmotCD* mutant.

**FIG 3:**
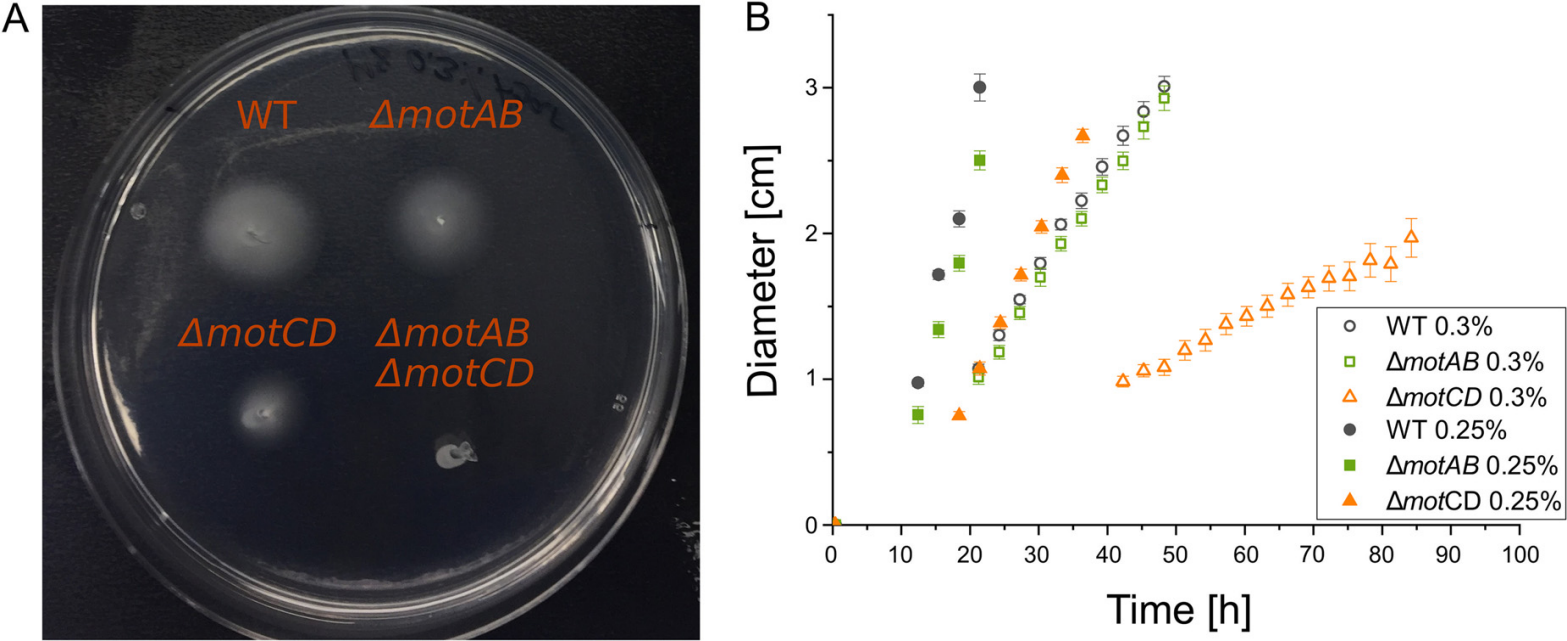
Spreading of bacterial cultures in swimming agar. (A) Spreading of the wild type, the single mutants *ΔmotAB* and *ΔmotCD*, and the double mutant *ΔmotAB ΔmotCD* in a 0.30% semisolid agar plate. The double mutant is nonmotile. (B) Time evolution of the diameter of wild-type (black), *ΔmotAB* mutant (green), and *ΔmotCD* mutant (orange) cultures in 0.25% (filled symbols) and 0.30% (open symbols) semisolid agar.

In their natural soil habitat, P. putida cells have to navigate through strongly confined, narrow spaces of complex, irregular geometry. To elucidate the role of the two stators with respect to swimming motility in such complex environments, we have investigated the swimming of P. putida in semisolid agar. Semisolid agar is a randomly structured, close-meshed, porous network filled with fluid (27). We injected cells into 0.25% and 0.30% semisolid agar and quantified the macroscopic spreading of the growing culture over time; see Fig. 3A. At a concentration of 0.25%, semisolid agar has a pore size in the range between 740 nm and 4,800 nm (27). As expected, cells spread more quickly in 0.25% than in 0.30% agar due to the difference in pore size; see Fig. 3B. For both agar concentrations, the *ΔmotAB* culture spreads almost as quickly as the wild type, whereas the *ΔmotCD* culture spreads considerably more slowly. This cannot be explained based on the observed swimming behavior in open fluids, where, in contrast to the spreading in agar, *ΔmotAB* cells are barely motile, and *ΔmotCD* cells swim at speeds similar to the wild type.

### The wild type and both stator mutants show similar speed distributions in agar, but wrapped mode formation is less frequent in the *ΔmotAB* mutant.

**FIG 4:**
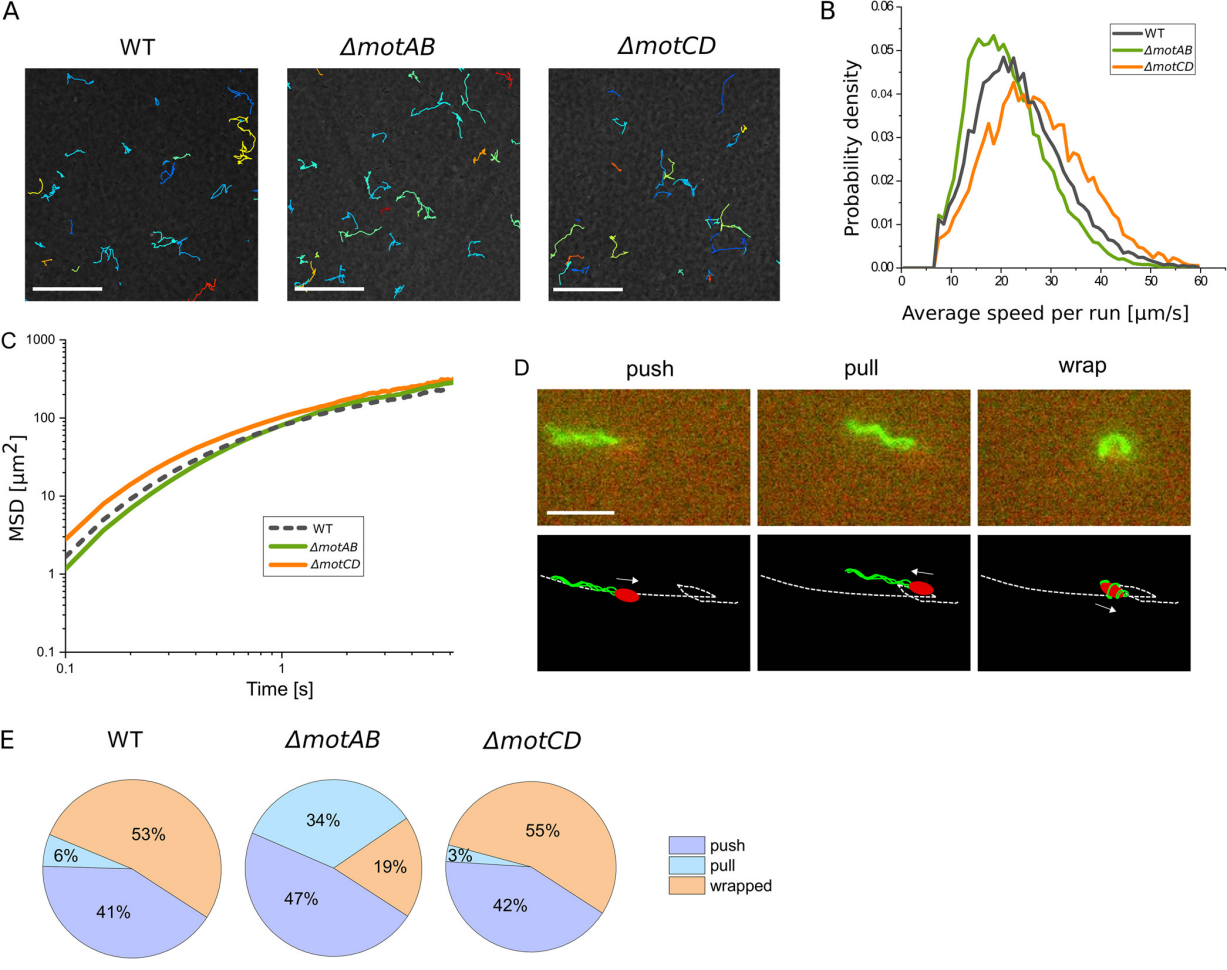
Single-cell motility in 0.25% swimming agar. (A) Swimming trajectories in semisolid agar. Scale bar, 100 μm. (B) Distribution of mean run speeds in semisolid agar. (C) Mean square displacement over time. (D) The three swimming modes in semisolid agar are shown for fluorescently labeled wild-type cells; see also the corresponding Movie S2 in the supplemental material. The arrows indicate the swimming direction. Scale bar, 5 μm. (E) Pie charts showing the frequency of swimming modes. We manually analyzed 327 runs for the wild type, 241 runs for the *ΔmotAB* mutant, and 189 runs for the *ΔmotCD* mutant.

To understand the different spreading efficiencies of wild-type and mutant strains in semisolid agar, we imaged and analyzed the motility of single cells in the agar matrix. Due to the narrow fibrous meshwork, swimming in semisolid agar differs from swimming in an open aqueous environment; see Movie S1 for an example. In particular, the length of straight runs is restricted to the free path length set by the geometry of the porous medium, resulting in more frequent interruptions of the runs as cells collide with or get stuck in the meshwork and have to reorient to escape. Figure 4A shows a selection of the longest swimming trajectories that we extracted from phase-contrast imaging experiments of cells swimming in semisolid agar. Using an adapted version of our MATLAB-based cell-tracking software, we determined the swimming speeds of the runs and the MSD for the three different strains in agar (Fig. 4B and C). Comparing the speed distributions of the three strains, it turns out that the differences between them are less pronounced in agar than in aqueous fluid, where the mean speed is much lower for the *ΔmotAB* mutant than for the wild type and the *ΔmotCD* mutant strain (Table 1). In the agar, in contrast, the mean speeds are much closer, with the *ΔmotCD* mutant swimming slightly more quickly and the *ΔmotAB* mutant slightly more slowly than the wild type. The mean speed per run is 23.85 *μ*m/s ± 0.05 μm/s for the wild-type, 21.44 μm/s ± 0.04 μm/s for the ΔmotAB mutant, and 27.18 μm/s ± 0.12 μm/s for the *ΔmotCD* mutant (please note that here the speed values are calculated for the run episodes only). Based on this observation, we cannot explain the slower spreading of the *ΔmotCD* mutant than the *ΔmotAB* and wild-type cultures in agar. However, the observation that the *ΔmotAB* mutant is almost nonmotile in liquid but swims as quickly as the wild type and the *ΔmotCD* mutant in agar indicates an increased recruitment of the MotCD stator to the motor under these conditions.

10.1128/mbio.02182-22.5 MOVIE S1 Comparison of swimming in liquid and swimming in agar. Download Movie S1, AVI file, 2.6 MB. Copyright © 2022 Pfeifer et al. 2022 Pfeifer et al. https://creativecommons.org/licenses/by/4.0/ This content is distributed under the terms of the Creative Commons Attribution 4.0 International license.

10.1128/mbio.02182-22.6 MOVIE S2 Dual-color fluorescence imaging of wild-type swimmer in agar. Download Movie S2, AVI file, 1.7 MB. Copyright © 2022 Pfeifer et al. 2022 Pfeifer et al. https://creativecommons.org/licenses/by/4.0/ This content is distributed under the terms of the Creative Commons Attribution 4.0 International license.

To determine the frequencies of the push, pull, and wrapped modes during swimming in semisolid agar, we labeled the flagella and the cell body with two different fluorescent dyes. Using dual-color fluorescence microscopy, we simultaneously imaged their position and relative orientation to identify the swimming mode of each run for the wild type and *ΔmotAB* and *ΔmotCD* mutant strains swimming in semisolid agar (Fig. 4D and Movies S2 to S4). The staining experiments revealed an increased wrapped mode formation for all three strains in comparison to swimming in aqueous fluid (Fig. 4E). Surprisingly, also, in semisolid agar, the distributions of swimming modes for the wild-type and the *ΔmotCD* mutant are similar, while a different distribution with a smaller portion of the wrapped mode is observed for the *ΔmotAB* mutant. This is consistent with the observations in viscous fluids but does not explain the different macroscopic spreading dynamics of the *ΔmotCD* culture compared to the wild-type and *ΔmotAB* cultures in semisolid agar (Fig. 3A).

### Cluster formations of the *ΔmotCD* mutant in semisolid agar result in fewer motile cells, thereby decreasing spreading of the culture.

**FIG 5:**
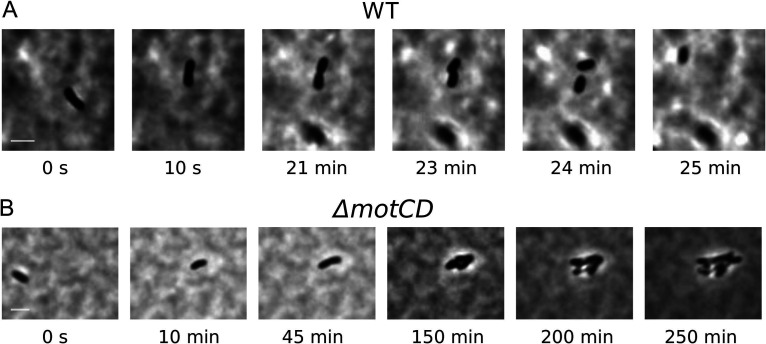
Cell division of trapped cells in semisolid agar. (A) Wild-type cells move on after division. (B) *ΔmotCD* mutant cells remain sessile and form growing clusters. See also the corresponding Movies S5 and S6 in the supplemental material. Scale bar, 5 μm.

In contrast to the wild-type and the *ΔmotAB* cultures, the number of motile cells of the *ΔmotCD* mutant strain decreased over time in the swimming agar assay. Instead, we noticed the formation of growing clusters of immobile cells. To analyze the cluster formation in more detail, we recorded the *ΔmotCD* culture for several hours and compared it to similar imaging experiments with wild-type and *ΔmotAB* cultures in semisolid agar. These recordings show that motile cells occasionally stop before they divide, most likely because they get trapped. For wild-type and *ΔmotAB* mutant cells, at least one, but often both, daughter cell moves on after division (Fig. 5A). In contrast, the *ΔmotCD* mutant cells mostly do not become motile again after division, so both daughter cells remain immobile. These cells are still alive, and by further divisions of the daughter cells, the above-mentioned cell clusters are formed (Fig. 5B). Cluster formation of the *ΔmotCD* mutant cells was more pronounced in 0.3% agar. Here, the daughter cells remained immobile after most of the cell divisions we observed. In 0.25% agar, in addition to the cluster-forming division events, also, cell divisions resulting in motile daughter cells were seen. Thus, the number of motile *ΔmotCD* cells was higher in 0.25% than in 0.3% agar but still lower than in the cultures of the wild-type and *ΔmotAB* mutant strains, resulting in a strongly reduced spreading of the *ΔmotCD* culture (Fig. 3).

10.1128/mbio.02182-22.7 MOVIE S3 Dual-color fluorescence imaging of *ΔmotAB* mutant in agar. Download Movie S3, AVI file, 6.2 MB. Copyright © 2022 Pfeifer et al. 2022 Pfeifer et al. https://creativecommons.org/licenses/by/4.0/ This content is distributed under the terms of the Creative Commons Attribution 4.0 International license.

10.1128/mbio.02182-22.8 MOVIE S4 Dual-color fluorescence imaging of *ΔmotCD* mutant in agar. Download Movie S4, AVI file, 3.3 MB. Copyright © 2022 Pfeifer et al. 2022 Pfeifer et al. https://creativecommons.org/licenses/by/4.0/ This content is distributed under the terms of the Creative Commons Attribution 4.0 International license.

10.1128/mbio.02182-22.9 MOVIE S5 Cell division of trapped wild-type cell. One frame was made every 50 seconds. Download Movie S5, AVI file, 0.7 MB. Copyright © 2022 Pfeifer et al. 2022 Pfeifer et al. https://creativecommons.org/licenses/by/4.0/ This content is distributed under the terms of the Creative Commons Attribution 4.0 International license.

10.1128/mbio.02182-22.10 MOVIE S6 Cell division of trapped *ΔmotCD* mutant cell forming a cell cluster. One frame was made every 20 seconds. Download Movie S6, AVI file, 1.3 MB. Copyright © 2022 Pfeifer et al. 2022 Pfeifer et al. https://creativecommons.org/licenses/by/4.0/ This content is distributed under the terms of the Creative Commons Attribution 4.0 International license.

## DISCUSSION

We examined the swimming motility of wild-type and stator mutant cells of the soil bacterium P. putida in different environments. In homogeneous fluids, the MotAB stator is crucial to promote swimming motility, so the movement of *ΔmotAB* mutant cells in aqueous fluids is strongly impaired. In contrast, in a heterogeneous, structured agar environment, both stators can promote swimming motility, but the MotCD stator, in addition, prevents cells from forming sessile clusters. Taken together, the two stators ensure robust swimming motility of P. putida under different environmental conditions.

Along with the strong swimming deficiencies in *ΔmotAB* mutant cells, also, the ratio of CCW to CW episodes of flagellar rotation is altered. While the wild type and the *ΔmotCD* mutant show approximately equal ratios of CCW (push) and CW (pull and wrapped) swimming modes, only 25% of the *ΔmotAB* mutant cells exhibited CW flagellar rotation (only pull, no wrapped mode) when swimming under low-viscosity conditions (Fig. 1D). For E. coli, it is known that CW-rotating motors generate a lower torque at intermediate load than CCW-rotating motors (28), which may be due to differences in stator-rotor interactions (6). If the same is true for P. putida, the increased portion of CCW runs may indicate that, under conditions where swimming motility is strongly impaired, cells adapt their locomotion strategy such that they preferentially rely on CCW rotation of their flagella to generate sufficient torque for propagation. Alternatively, MotAB knockout might directly influence the chemotaxis pathway, leading to an altered CW bias. This is supported by observations in P. aeruginosa showing that MotC interacts with the diguanylate cyclase SadC, which may lead to increased production of the key motility regulator cyclic diguanylate (29). Furthermore, for E. coli, it was shown that flagellar switching may also occur independently of CheY phosphorylation in the chemotaxis pathway and is sensitive to external load and the energy input provided by the stators (30, 31).

For the *ΔmotAB* mutant, not only the relative frequencies of CCW and CW swimming modes are altered, but also, the ratio of pull and wrapped modes, which may both occur during CW rotation of the flagellar motors, is drastically changed. For wild-type and *ΔmotCD* mutant cells, both pull and wrapped mode runs are frequently observed (Fig. 1D), with an increasing portion of wrapped runs for higher viscosities (Fig. 2), similar to previous observations in S. putrefaciens (22). In contrast, under low-viscosity conditions, the *ΔmotAB* mutant does not exhibit any wrapped mode formation at all (Fig. 1D). Only for increased viscosity and in an agar meshwork, a low fraction of wrapped runs is observed for this mutant strain (Fig. 2 and Fig. 4E). Thus, wrapped mode formation is not restricted to one type of stator. Also, the MotCD stator is sufficient to form the wrapped mode under increased load, although less efficiently. It was previously conjectured that wrapped mode formation results from an instability of the pulling bundle that is triggered by an increase in motor torque (19, 22). Numerical simulations of lophotrichously flagellated bacteria have confirmed this torque-dependent filament wrapping (26). We thus assume that, under low-viscosity conditions, *ΔmotAB* mutant cells are not able to form the wrapped mode because not enough torque is generated by the MotCD stators that are less active in fluids, cf. also the low swimming speeds of the *ΔmotAB* mutant cells. Under increased load, presumably more MotCD stators are recruited to the motor, increasing the torque to such an extent that occasional wrapped mode formation can be observed. This remodeling in the motor assembly by changing the stator composition at the motor is characteristic of bacteria with multiple stators. For example, in P. aeruginosa, fewer MotCD stators bind to the motor during operation in a low-viscosity environment (14).

Our findings for P. putida differ from earlier observations reported for *ΔmotAB* and *ΔmotCD* mutants in P. aeruginosa. For swimming in fluids, the speed of the *ΔmotAB* knockout in P. aeruginosa is only slightly reduced compared to the wild-type (12), in contrast to the strong effect we observed for the P. putida *ΔmotAB* mutant (Table 1). Furthermore, for P. aeruginosa, it was shown that under increased load on the flagella, more MotCD units are recruited to the motor (14). An increased viscosity thus only leads to a decrease in motility for the *ΔmotAB* mutant and the wild-type, while it abolishes motility in the *ΔmotCD* mutant for 15% Ficoll (13). In contrast, for P. putida, we observe that, under high-viscosity conditions, the *ΔmotAB* mutant exhibits only little wrapped mode formation, and its swimming speed remains low, while *ΔmotCD* cells are comparable to the wild type in terms of wrapped mode formation and swimming speeds (Fig. 2 and Table 1). Consequently, for P. putida, the MotAB stator performs better, independent of the Ficoll concentration. Stator remodeling in response to increased Ficoll concentrations thus seems less pronounced in P. putida than in P. aeruginosa.

Only when changing the environmental conditions to a heterogeneous agar meshwork, we see that the *ΔmotAB* mutant can outperform the *ΔmotCD* mutant by spreading more quickly in semisolid agar (Fig. 3). Interestingly, P. putida distinguishes between the two environments of higher viscosity due to Ficoll addition and the heterogeneous agar meshwork. Naively, one could expect that in both cases, mechanosensing results in similar stator remodeling. However, it seems that only in the agar, the MotCD stator is recruited to such an extent that the *ΔmotAB* mutant swims and spreads as quickly as the wild type in the soft agar. We thus conclude that the strong confinement and close interaction with the agar network, where cells get frequently trapped, result in the mechanical stresses that are required for the corresponding stator remodeling.

The similar spreading of the *ΔmotAB* mutant and wild-type cells, as well as the decreased spreading of the *ΔmotCD* mutant, agrees with earlier observations in P. aeruginosa (12). However, the slower spreading of the P. putida *ΔmotCD* culture is not caused by a decreased swimming speed. Surprisingly, the *ΔmotCD* mutant swims at similar speeds or slightly more quickly than the *ΔmotAB* and wild-type strains (Fig. 4B), but fewer motile cells are observed due to the formation of sessile clusters (Fig. 5). The mechanism behind the cluster formation is yet unknown. It may be related to the surface-sensing properties of MotAB, which were observed for P. aeruginosa (32). We assume that cluster formation is the main reason for the slower spreading of the *ΔmotCD* culture. Effectively, clustering decreases the amount of newly formed motile daughter cells, which will influence cell culture spreading (33). Specifically, we assume that the observed linear increase in culture radius (Fig. 3) results from an interplay of reproduction and motility, as was previously proposed (27), where a reduced growth rate of motile cells due to clustering will lead to a decreased slope in the linear time profile of the culture radius.

Our observation that the *ΔmotCD* mutant swims more quickly than the *ΔmotAB* mutant under all tested conditions indicates that the MotAB stator generates a higher total torque than the MotCD stator. Whether this is due to more efficient torque generation of the individual stators or caused by different numbers of stator units associated with the motor cannot be decided from our data. In addition, the intermediate swimming speed of the wild type in agar suggests that the flagellar motor can bind MotAB and MotCD stators simultaneously (Fig. 4B). This would be in agreement with P. aeruginosa, where coexistence of the two stator sets at the flagellar motor has already been shown (34).

The natural habitat of P. putida is a heterogeneous, crowded soil environment. While earlier experiments in larger confined spaces, such as between parallel plates or cylindrical obstacles, revealed only slight modulations of the swimming pattern known from uniform aqueous liquid (35, 36), our present experiments in agar can be seen as a first step to mimicking a more complex, dense environment. The increased wrapped mode formation in agar that we observed for the wild type, as well as for both stator mutant strains, agrees with earlier observations that the wrapped mode typically occurs under external confinement and most likely serves as a strategy of flagellated cells to escape from mechanical traps (22, 23) or to enhance environmental spreading (37). It also fits the prediction that P. putida preferentially uses this slow swimming mode to navigate its natural environment (20, 26). How bacteria sense mechanical confinement and trapping remains an open question that will be addressed in subsequent studies. Furthermore, we aim to investigate, in future experiments, how the pore size and other geometric parameters of the environment influence the stator composition at the motor and the overall spreading dynamics in inhomogeneous environments. For this purpose, not only agar substrates will be used but also custom-made microfluidic chambers and other soft materials, such as hydrogels, that have already been successfully applied to describe the motility of E. coli in a complex, confined matrix (38, 39). In addition, for a better understanding of stator remodeling, we are planning to investigate the stator recruitment with labeled stators and determine the expression levels of the two stators under different environmental conditions.

## MATERIALS AND METHODS

### Cell culture.

We used the strain P. putida KT2440 FliC*_S_*_267_*_C_*. In a previous study, the flagellin protein FliC was genetically modified by exchanging serine 267 with a cysteine in order to fluorescently label the flagella. It was shown that this exchange does not influence motility (19). We refer to this strain as the wild type. P. putida was grown in shaking culture at 30°C, and the required E. coli strains for cloning were grown at 37°C. For cloning and the swimming agar assay, cells were inoculated in lysogeny broth. For flagellar staining experiments, cells were grown overnight in tryptone broth (10 g/L tryptone [Applichem], 5 g/L NaCl).

### Construction of vectors and strains.

All required oligonucleotides and plasmids used in this study are listed in Table S2 in the supplemental material. To knock out the stator genes *motAB* or *motCD*, for each stator, its up- and downstream regions were amplified and cloned into the suicide vector pNPTS138-R6K via Gibson assembly and transformed into E. coli DH5αλpir-competent cells. The recombinant vector was transformed into the donor cells of E. coli WM3064 and further via conjugation to P. putida. In P. putida, we selected for sequential double-homologous recombination (40). The reintroduction of the knocked-out genes for complementation was performed in the same way: the gene was reintegrated in the corresponding deletion strains into the native locus.

10.1128/mbio.02182-22.4 TABLE S2 Oligonucleotides and plasmids used for molecular cloning. Download Table S2, PDF file, 0.1 MB. Copyright © 2022 Pfeifer et al. 2022 Pfeifer et al. https://creativecommons.org/licenses/by/4.0/ This content is distributed under the terms of the Creative Commons Attribution 4.0 International license.

### Swimming experiments in fluids.

Fluid experiments were carried out in μ-Slide VI 0.1 channels (Ibidi). To increase viscosity, 10%, 15%, and 20% (wt/vol) Ficoll 400 was added. This corresponds to viscosities of 5, 10, and 18 cP (22, 41).

### Staining experiments.

Flagella were stained with the fluorescent dye Alexa 488 C_5_ maleimide (Thermo Fisher Scientific). The staining was carried out as published before in Hintsche et al. (19). For agar experiments, we additionally stained the cell body with 10 μL FM 4-64 (Thermo Fisher Scientific; 1 μg/μL in dimethyl sulfoxide [DMSO]). It was added before the last washing step. Previous experiments showed that the double staining does not affect the swimming modes.

### Swimming agar assay.

The swimming agar assay was carried out according to Ha et al. (42) with 0.3% and 0.25% semisolid agar. In short, M8 (42.4 g/L disodium phosphate dihydrate, 15 g/L monopotassium phosphate, and 2.5 g/L sodium chloride) agar plates with 0.2% glucose, 0.5% Casamino Acids, and 1 mM magnesium sulfate as final concentration were poured into petri dishes. Plates solidified over 4 h. A small volume of cells was injected into the agar. Recordings of stained cells were carried out after an additional 4 h. After this time, the cells adapted to the new environment and spread away from the injection point where the agar was destroyed. We could not wait for more than 4 h because, afterward, we barely found any stained cells, as newly formed flagella, which are built during cell division, are not stained. Phase-contrast recordings were performed the next day. For the analysis of the culture spreading in semisolid agar, standard petri dishes were used. For analysis of single cells, FluoroDish cell culture dishes with glass bottoms were used.

### Microscopy.

Microscopy was carried out with an inverted microscope (Olympus IX71) with a blue LED as excitation source (4.8-W optical output power, 470 nm) for fluorescence microscopy and a white LED as the light source for phase contrast. The Orca-Fusion BT digital complementary metal oxide semiconductor (CMOS) camera from Hamamatsu was used for recordings together with the Hokawo software (Hamamatsu). Phase contrast was recorded 30 μm over the bottom surface with a 20× UPLFLN-PH objective (Olympus) and 20 frames per second to analyze single-cell swimming motility. To identify cluster formation of the *ΔmotCD* mutant in the swimming agar assay, we recorded the culture for 15 h with 3 frames per minute and compared it to similar imaging experiments of wild-type and *ΔmotAB* cultures. Fluorescence images were recorded close to the surface with 100 frames per second using a 60× UPLFLN-PH objective (Olympus). For the double-fluorescence staining of the flagella and the cell body in the agar experiments, the W-View Gemini image-splitting optics (Hamamatsu) were used. Fluorescence images were processed with ImageJ as described in Hintsche et al. (19). The frequency of swimming modes was determined by counting the number of observed modes.

### Processing with MATLAB.

For segmentation and cell tracking, the code described in Theves et al. (18) was used, which is based on Crocker and Grier (43). For measurements in bulk, the tested filter parameters also remain the same. Trajectories with an average velocity of <10 μm/s, a displacement of <5 μm, or a length of less than 2 s, as well as highly curved trajectories were filtered out; see reference 18. For measurements in agar, on the other hand, only very short trajectories (<1.5 s) and trajectories with very low displacement (<1 μm) were filtered out since the velocities in agar are generally lower and bacteria do not move as much and as straight as in the bulk. To identify runs (Fig. 4), we used two conditions. All the phases of a track with a current speed that is below the threshold value of 7 μm/s were not classified as runs. These phases may be called traps, following the wording of Bhattacharjee and Datta (38). As a second condition, time points of the tracks were excluded from run phases if they were located between two traps, where the start point of the second trap was less than 2 μm away from the endpoint of the previous trap. Two traps that are very close to each other thus merge into a single larger trap. This second condition had to be introduced because, from visual inspection, it became obvious that larger traps were frequently split into several smaller trap events when only the first condition was used. All time points that were not traps were classified as runs.
