# Supplementary material for: Role of the Two Flagellar Stators in Swimming Motility of Pseudomonas putida
Source: mBio. 2022 Nov 21;13(6):e02182-22. doi: 10.1128/mbio.02182-22 (PMC9765564; doi:10.1128/mbio.02182-22)
Supplement: FIG S1 [file mbio.02182-22-s0001.pdf]

Query: P. putida motA Query ID: lcl|Query_49295 Length: 283

>P. aeruginosa motA
Sequence ID: Query_49297 Length: 283
Range 1: 1 to 283

Score:466 bits(1199), Expect:1e-172,
Method:Compositional matrix adjust.,
Identities:230/283(81%), Positives:254/283(89%), Gaps:0/283(0%)

```
Query  1    MAKIIGIIVVFASVLGGYVLSHGKIAALIQPFEVLIIGGAAFGAFLQANPGHMTMHVIKK  60
            M+KIIGIIVVFASVLGG++LS GKI A+IQPFEVLIIGGAA GAFLQ+NPG   M V+KK
Sbjct  1    MSKIIGIIVVFASVLGGFLLSGGKIGAIIQPFEVLIIGGAALGAFLQSNPGSTFMVVLKK  60

Query  61   SMKMFGSRFSHAFYLEVLGLVYEILNKSRREGMMAIEADIEDAAASPIFAKYPTVLADER  120
            + KMF +RF+  +YLEVLG++YEILNKSRREGMMAIEADIED AASPIF+KYP VL DER
Sbjct  61   APKMFSNRFTQTYYLEVLGMLYEILNKSRREGMMAIEADIEDPAASPIFSKYPGVLKDER  120

Query  121  MTAFVCDYLRIMSTGNMAPHELEGLFDMELLSMKEELEHPSHAVTGIADGMPGFGIVAAV  180
            MTA+VCDYLRIMS+GNMAPHELEGLFDMEL S+KE+LEHPSHAVT +AD +PGFGIVAAV
Sbjct  121  MTAYVCDYLRIMSSGNMAPHELEGLFDMELSSLKEDLEHPSHAVTKVADALPGFGIVAAV  180

Query  181  LGIVVTMASLGDGDQAAIGMHVGAALVGTFFGILAAYGFFGPLAKCLEHDAKEELNLYES  240
            LGIV+TMA LG+G QA IG HV AALVGTF GILAAYGF GPLA  LEHDAKEELNL+E+
Sbjct  181  LGIVITMALLGEGSQAEIGHHVAAALVGTFLGILAAYGFVGPLAGALEHDAKEELNLFEA  240

Query  241  IKASLVASASGMPPSLAVEFGRKVLYPKHRPSFAELEQAVRGR  283
            IKA LVASASGMPPSLAVEFGRKVL P HRP+FAELEQAVRGR
Sbjct  241  IKACLVASASGMPPSLAVEFGRKVLLPAHRPTFAELEQAVRGR  283
```

Query: P. putida motB Query ID: lcl|Query_39849 Length: 346

>P. aeruginosa motB
Sequence ID: Query_39851 Length: 347
Range 1: 1 to 345

Score:459 bits(1181), Expect:9e-168,
Method:Compositional matrix adjust.,
Identities:250/357(70%), Positives:292/357(81%), Gaps:25/357(7%)

```
Query  1    MENNQPIIVKRVKRFGGGHHGGAWKIAFADFATAMMAFFLVLWLLSTATPEQKIAIAGYF  60
            M+NNQPIIVKRVKR+  GHHGG+WKIAFADFATAMMAFFLVLWLLS+ATPEQK AI+GYF
Sbjct  1    MDNNQPIIVKRVKRYAAGHHGGSWKIAFADFATAMMAFFLVLWLLSSATPEQKKAISGYF  60

Query  61   QDPIGFSESGTPYVIDLGGSPEMAPDKTINPEVKTEP----TQQSP---TQLSKDQVETM  113
            QDPIGFSES +PYVIDLGG+P  APDKT+NP+V+ +P    ++ SP     Q++ DQ E +
Sbjct  61   QDPIGFSESASPYVIDLGGTPTPAPDKTLNPQVQAQPDSNESRISPEQDHQVNADQAENL  120

Query  114  AEQVERERLELLLQELQNKVEENPQLQKFKDQILFEITQDGLRIQIMDAENRPMFDIGSA  173
            AEQVERERL LLLQELQNKV+ENP L+ FKDQI FEIT+DGLRIQI+DA NRPMFD+GSA
Sbjct  121  AEQVERERLALLLQELQNKVDENPMLKDFKDQIHFEITRDGLRIQIVDAANRPMFDLGSA  180

Query  174  RLQPYFEDILLAMADTIKAVPNKVSISGHTDAKPYAGTGEYGNWELSANRANAARRALVA  233
            RLQPYFEDILLAMA+TI+ VPNK+SISGHTDAKPYAG G++GNWELSANRANAARRALVA
Sbjct  181  RLQPYFEDILLAMAETIRQVPNKISISGHTDAKPYAGNGDFGNWELSANRANAARRALVA  240

Query  234  GGYPDGQVARVVGYASSSLFDRKDPFNPVNRRIDIIVLTKKAQRNIEGEQGAPETPPAAP  293
            GGYP+GQ+A+VVGYAS+ LFD KDP NPVNRRIDI+VLT+KAQ+ IEGE GAPE    A
Sbjct  241  GGYPEGQIAQVVGYASARLFDDKDPLNPVNRRIDIVVLTRKAQKAIEGETGAPEASAPAA  300

Query  294  -----TAPAAPASGAAPGASAPADPGATEQAPMQPRELRQKLNIFED-GTLKMDEAK  344
                   PAA A  A    S PAD            E+++KLN+F+D G+LK+++ K
Sbjct  301  APGEQPKPAAEAPAAGAQPSLPAD------------EVQKKLNLFDDGGSLKLEQIK  345
```

Query: P. putida motC Query ID: lcl|Query_52511 Length: 246

>P. aeruginosa motC
Sequence ID: Query_52513 Length: 246
Range 1: 1 to 246

Score:417 bits(1072), Expect:1e-154,
Method:Compositional matrix adjust.,
Identities:205/246(83%), Positives:229/246(93%), Gaps:0/246(0%)

```
Query  1    MDVLSLIGLILAFVAIVGGNFLEGGHVGALVNGPAALIVLGGTLAAALLQSPLTSFKRAL  60
            MDVLSL+G+ILAFVAIVGGNFLEGGH GAL+NGPAALIV+GGTLAAALLQ+P+   KR L
Sbjct  1    MDVLSLVGIILAFVAIVGGNFLEGGHAGALLNGPAALIVIGGTLAAALLQTPVNVLKRGL  60

Query  61   QILRWILFPPRVDLAGGIDRVVNWSLTARKEGLLGLEGVADSEPDPYARKGLQLLVDGAE  120
             +L W+ FPPR DL+GGIDR+V+WS+TARKEGLLGLE +AD+EPDPYARKGLQLLVDGAE
Sbjct  61   GMLGWVFFPPREDLSGGIDRIVSWSMTARKEGLLGLESIADAEPDPYARKGLQLLVDGAE  120

Query  121  PEAIRSILEVDLLTQEGRDIQAAKVFESMGGYAPTIGIIGAVMGLIHVMGNLADPSQLGN  180
            PE IRSILEVDL TQE RD+QAAKVFESMGGYAPTIGIIGAVMGLIHVMGNLA+P+QLG+
Sbjct  121  PEVIRSILEVDLFTQESRDLQAAKVFESMGGYAPTIGIIGAVMGLIHVMGNLANPAQLGS  180

Query  181  GIAVAFVATIYGVASANLVLLPVASKLKAIVMRQSRYREMLLEGLLSIAEGENPRSIELK  240
            GIAVAFVATIYGV  ANL+LLP+ +KLK +V+RQSRYREMLLEGLLSIAEGENPRSIELK
Sbjct  181  GIAVAFVATIYGVGFANLLLLPIGNKLKTLVLRQSRYREMLLEGLLSIAEGENPRSIELK  240

Query  241  LQGFME  246
            LQGFM+
Sbjct  241  LQGFMD  246
```

Query: P. putida motD Query ID: lcl|Query_64363 Length: 285

>P. aeruginosa motD
Sequence ID: Query_64365 Length: 296
Range 1: 2 to 275

Score:403 bits(1035), Expect:2e-147,
Method:Compositional matrix adjust.,
Identities:197/280(70%), Positives:229/280(81%), Gaps:6/280(2%)

```
Query  1    MRRRRHTEEHENHERWLVSYADFITLLFAFFVVMYSISSINEGKYKVISQALLGVFNDPE  60
             RRRRH EEHENHERWLVSYADFITLLFAFFVVMYSISSINEGKYK++S+ L GVFN P+
Sbjct  2    QRRRRHQEEHENHERWLVSYADFITLLFAFFVVMYSISSINEGKYKILSETLTGVFNQPD  61

Query  61   RSMKPIPIGDEQPLSVRPAEPLVKDSEQTEAGLAATNVDPLKTISDDVRDAFGDLINSDQ  120
            RS+KPIPIGDE+P +  P    V++       A  A    D L+ I+D VRDAFGDLI SDQ
Sbjct  62   RSLKPIPIGDERPRTTEPERTSVEEQPSDNAASA----DSLERIADSVRDAFGDLIASDQ  117

Query  121  MTVRGNELWIEIELNSSLLFGSGDAMPSDKAFAIIEKVASILKPFANPVHVEGFTDNLPI  180
            ++VRGNELWIEI LNSSLLF SGDA+P+D AF I+EKVA IL P+ NP+HVEGFTD++PI
Sbjct  118  LSVRGNELWIEITLNSSLLFPSGDALPNDAAFDIVEKVAKILAPYKNPIHVEGFTDDVPI  177

Query  181  RTAQYPTNWELSSARAASIVRLLAMEGVNPARMASVGYGEYQPVASNDTAEGRARNRRVV  240
             + +YPTNWELS+ARAASIVRLL  +GV P+RMA+VGYGE+QPVA N +AEGRA+NRRVV
Sbjct  178  HSPRYPTNWELSAARAASIVRLLGNDGVEPSRMAAVGYGEFQPVADNASAEGRAKNRRVV  237

Query  241  LVISRNLEVRRSLTGSGSANATPDAALRRAGTQSAPAIQA  280
            LVISRNLEVRRS++G GS  A PD+ALR AG  SA   QA
Sbjct  238  LVISRNLEVRRSVSGVGSGKAQPDSALRHAG--SAAGTQA  275
```
