# Supplementary figures and images for: Role of the Two Flagellar Stators in Swimming Motility of Pseudomonas putida
Source: mBio. 2022 Nov 21;13(6):e02182-22. doi: 10.1128/mbio.02182-22 (PMC9765564; doi:10.1128/mbio.02182-22)

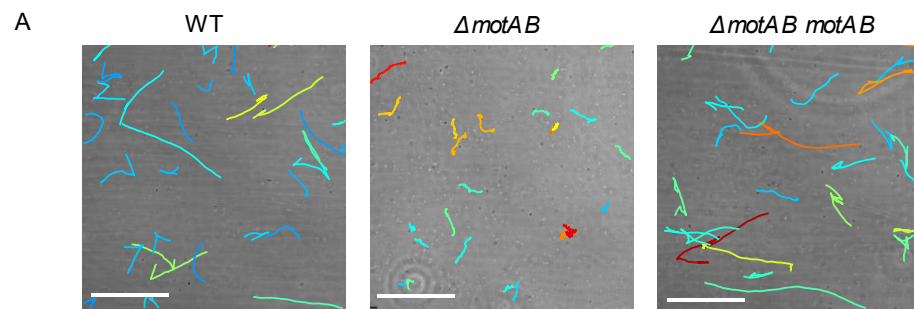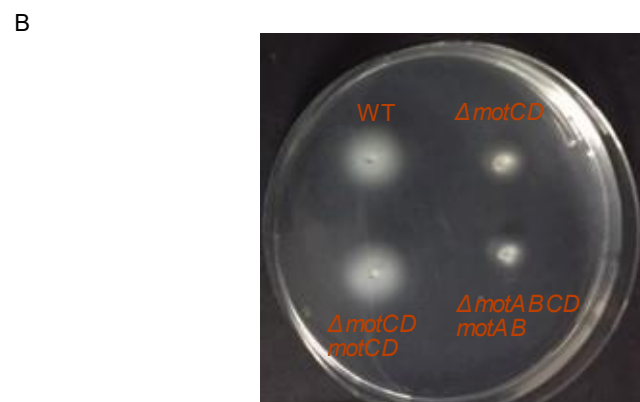

Supplement: FIG S2 [file mbio.02182-22-s0002.pdf]
