# Supplementary material for: Role of the Two Flagellar Stators in Swimming Motility of Pseudomonas putida
Source: mBio. 2022 Nov 21;13(6):e02182-22. doi: 10.1128/mbio.02182-22 (PMC9765564; doi:10.1128/mbio.02182-22)
Supplement: TABLE S1 [file mbio.02182-22-s0003.pdf]

TABLE S1. Frequency of swimming modes for different Ficoll concentrations.

For each experiment the sample size is listed. For the *ΔmotAB* mutant it is smaller due to the decreased motility. However, for 0% and 20% Ficoll we analysed a bigger amount of measurements to get a comparable sample size.

Ficoll 400	WT	*ΔmotAB*	ΔmotCD
0%	Push: 50%<br>Pull: 17%<br>Wrap: 33%<br>#Runs: 441	Push: 75%<br>Pull: 25%<br>Wrap: 0%<br>#Runs: 216	Push: 54%<br>Pull: 19%<br>Wrap: 27%<br>#Runs: 268
10%	Push: 52%<br>Pull: 2%<br>Wrap: 46%<br>#Runs: 400	Push: 62%<br>Pull: 35%<br>Wrap: 3%<br>#Runs: 29	Push: 60%<br>Pull: 6%<br>Wrap: 34%<br>#Runs: 325
15%	Push: 55%<br>Pull: 1%<br>Wrap: 44%<br>#Runs: 127	Push: 51%<br>Pull: 34%<br>Wrap: 15%<br>#Runs: 47	Push: 56%<br>Pull: 2%<br>Wrap: 42%<br>#Runs: 202
20%	Push: 62%<br>Pull: 1%<br>Wrap: 37%<br>#Runs: 284	Push: 65%<br>Pull: 32%<br>Wrap: 3%<br>#Runs: 114	Push: 62%<br>Pull: 0%<br>Wrap: 38%<br>#Runs: 176
