# Supplementary material for: Role of the Two Flagellar Stators in Swimming Motility of Pseudomonas putida
Source: mBio. 2022 Nov 21;13(6):e02182-22. doi: 10.1128/mbio.02182-22 (PMC9765564; doi:10.1128/mbio.02182-22)
Supplement: TABLE S2 [file mbio.02182-22-s0004.pdf]

TABLE S2. Oligonucleotides and Plasmids used for molecular cloning.

	**Name**	**Sequence/ Insert**	**Purpose**
**Oligonucleotides**	motAB_KO_Gibs_fwd	GCCAAGCTTCTCTGCAGGATATCTGGATCCTTGCAGGTACAGCG	Construction of pNPTS138-R6KT-motAB_KO to knock-out the MotAB gene
	motAB_KO_Gibs_rv	GCCGAAGCTAGCGAATTCGTGGATCCCCGAACAACCATGGTCAA	
	motCD_KO_Gibs_fwd	CGCCAAGCTACGTAATACGACTCGCGGCCGCGGAAGCAATCGACCAGGCG	Construction of pNPTS138-R6KT-motCD_KO to knock-out the MotCD gene
	motCD_KO_Gibs_rv	GCTTCAATTGCACGGGCCCCACTAGTGGGTGCTGGTTTGCCGG	
	pVP17Gibs_fwd	AGCTTCTCTGCAGGATATCTGGATCCGCTGGCTGATCATCTGGAA	Construction of pNPTS138-R6KT-motAB to rescue the MotAB gene
	pVP17Gibs_rv	GCCGAAGCTAGCGAATTCGTGGATCCATGTCAGCCGCGACGACG	
	pVP18Gibs_fwd	AGCTTCTCTGCAGGATATCTGGATCCCAATTTCCCGGCGCCGAT	Construction of pNPTS138-R6KT-motCD to rescue the MotCD gene
	pVP18Gibs_rv	GCCGAAGCTAGCGAATTCGTGGATCCCATTCTGCCCTGGCGACT	
**Plasmids**	pNPTS138-R6KT	-	Suicide vector for in-frame deletion or integration
	pNPTS138-R6KT-motAB_KO	Upstream + Downstream region of motAB	Knockout MotAB
	pNPTS138-R6KT-motCD_KO	Upstream + Downstream region of motCD	Knockout MotCD
	pNPTS138-R6KT-motAB_complement	Upstream + *motAB* +Downstream region of motAB	Complementation MotAB
	pNPTS138-R6KT-motCD_complement	Upstream + *motCD* +Downstream region of motCD	Complementation MotCD
